# Use of Osteofasciocutaneous Fibular Free Flap and Radial Head Arthroplasty in Trauma for Limb Salvage and Continued Elbow Function

**DOI:** 10.1155/2018/8295736

**Published:** 2018-04-23

**Authors:** Catherine Kilmartin, Katharine D. Harper, Chirag Mehta, Joseph Thoder, Andrew Newman

**Affiliations:** ^1^Department of Surgery, Temple University School of Medicine, Philadelphia, PA, USA; ^2^Division of Orthopedic Surgery, Department of Surgery, Temple University of Medicine, Philadelphia, PA, USA; ^3^Division of Plastic Surgery, Department of Surgery, Temple University of Medicine, Philadelphia, PA, USA; ^4^Division of Plastic Surgery, Department of Surgery, Cooper University Hospital, Camden, NJ, USA

## Abstract

Reconstructive flaps have revolutionized the ability of surgeons to restore function and cosmesis for patients. While reconstructive flaps have been used to bridge large defects due to oncologic or congenital maladies necessitating large debridements, few cases have observed salvage flaps in traumas which provide additional challenges secondary to an injury trajectory. This case report details use of an osteofasciocutaneous fibular free flap and radial head prosthesis to restore forearm function in a 64-year-old female with a comminuted fracture of the proximal radius. The patient has sustained a 5.5 cm epiphyseal radial defect with an associated 20 × 15 cm overlying tissue defect after serial debridement. In review of the literature, only one nontraumatic case using a combined free flap and radial head prosthesis for proximal forearm defect to restore joint function has been reported. We suggest that, for proximal forearm fractures, this technique can be used to restore elbow joint function in limb salvage.

## 1. Introduction

Loss of elbow function leads to major morbidity secondary to a decreased ability to perform activities of daily living. Rotation of forearm and the range of motion of the elbow depend on the bicondylar articulation of the radius and ulnar with the humerus [1]. Case studies have demonstrated restorative free tissue flaps for distal forearm function; however, not many have investigated osteocutaneous free flaps in the acute trauma setting. Most of the reported cases for fibular osteocutaneous flaps to upper extremities were for nonunions, malignancy [2], osteomyelitis [2], or late reconstructions [3].

Studies have recommended the indications for osteocutaneous free flap transfers to the forearm be reserved for patients who have failed bone grafting or those with defects exceeding 6 cm [4]. One clinical trial demonstrated a fibular free tissue flap for a proximal radius fracture. At the 6-month follow-up, the patient had minimal limitation of forearm rotation and, at five years, he had returned to premorbid activities with comparable strength and motor function [3].

To our knowledge, only one study has been addressed using radial head prosthesis in conjunction with the osteocutaneous fibular flap to restore elbow stability and range of motion [5]. There are rare cases of elbow reconstruction with arthroplasty, with more reported cases of distal humeral fractures, proximal ulnar fractures, and interposition arthroplasty [6]. We present a patient with extensive upper extremity soft tissue damage and bone loss in the acute traumatic setting, where an osteofasciocutaneous free fibular flap with radial head arthroplasty allows for mobility and function of the forearm and elbow.

## 2. Case Report

**Figure 1:**
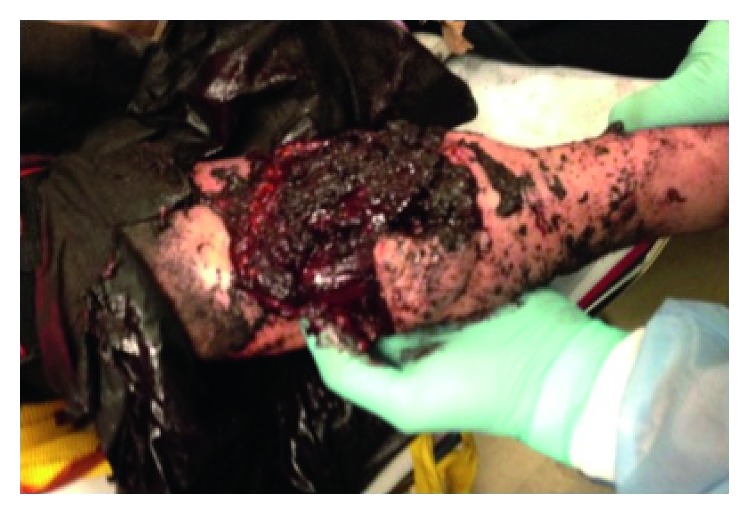
Injury at presentation in trauma bay, demonstrating gross contamination of the open fracture.

**Figure 2:**
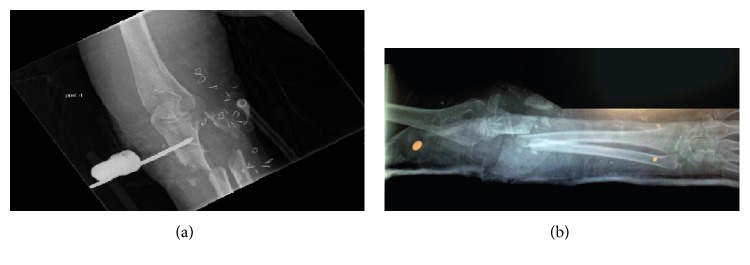
(a) AP X-ray after application of external fixator device demonstrating bony injury on initial injury after debridement. (b) AP X-ray showing the bony injury involving the ulna and radius, with a large amount of radial bone loss.

A 64-year-old female patient presented to the emergency department after a train derailment. Upon arrival, she was nonresponsive, and Advance Trauma Life Support protocol was initiated. She was intubated, and on further workup, she was found to have a right upper extremity- (RUE-) comminuted displaced fracture of proximal radius and ulna Gustilo grade IIIc open fracture with large degloving injury and gross contamination.  Figure 1 illustrates the large bony and subcutaneous tissue defect, and Figure 2(a) shows the X-ray of bony loss after application of an external fixator device while Figure 2(b) is the X-ray obtained in the trauma bay on initial arrival.

**Figure 3:**
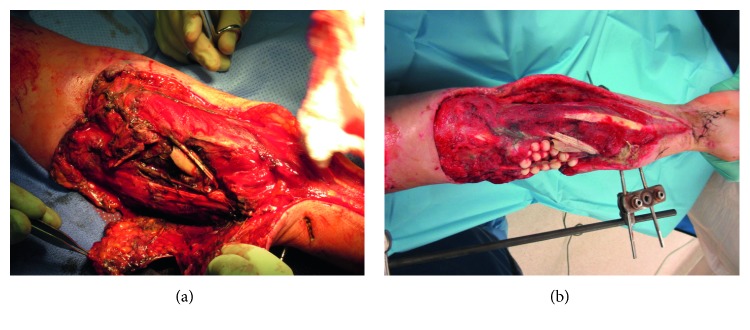
(a) The original debridement with the bony and soft tissue defect. (b) Antibiotic beads in the bony defect on figure fold with external fixator.

The patient was taken urgently to the operating room (OR) for right upper extremity washout, elbow-spanning external fixation, proximal ligation of the radial artery with angiogram secondary to substantial damage to the artery from the initial injury complex, right forearm fasciotomy, decompression of the carpal tunnel, and placement of wound VAC. Intraoperative angiogram demonstrated patent ulnar artery flow with an intact arch and backflow to the radial artery in the wrist. The soft tissue injury measured 15 cm × 10 cm, as seen in Figure 3(a), which shows the original debridement soft tissue and bony defect.  Figure 3(b) demonstrates the bony defect with antibiotic bead placement. On the first postoperative day, the patient was alert enough to undergo a neurovascular exam, where a posterior interosseous nerve (PIN) injury was noted.

The patient was taken back to the OR for multiple additional washouts and antibiotic bead exchange. On postoperative day (POD) 8, the patient had placement of ulnar intramedullary nail secondary to the only remaining skin bridge which was along the ulnar shaft and decision was taken to preserve the remaining skin bridge. Wound cultures from the initial surgery grew *Citrobacter fruendii*, *Serratia marcescens*, and *Enterococcus faecalis*, for which antibiotic regimen was adjusted based on susceptibilities, and the patient was treated for 6 weeks with amoxicillin and ciprofloxacin.

The patient's resultant defect consisted of a 5.5 cm radial epiphysis loss with an intact radial head and neck at the level of the elbow, a 20 × 15 cm overlying soft tissue defect, nearly circumferential, of the forearm involving her antecubital fossa. A repeat angiogram demonstrated flow through the ulnar artery with profuse flow to the midupper palmar arch and retrograde flow to the distal radius via the superficial palmar arch. She also had occlusion of the interosseous artery from its origin into the mid forearm with reconstitution in the distal forearm via collaterals.

In a multidisciplinary approach, she underwent an open reduction internal fixation (ORIF) of radius with a vascularized fibular osteocutaneous free flap to replace the proximal radius and overlying soft tissue. Radial head arthroplasty was performed at the proximal end of the fibular graft because the short segment of the native proximal radius remains.

The osteocutaneous fibular harvest was dissected in a standard fashion from the left lower extremity with a planned overlying skin paddle under sterile tourniquet control. The proximal osteotomy was completed so that the fibular would be reamed to fit the radial arthroplasty system while the flap was still vascularized. The fibular length after distal osteotomy was 6 cm. The radial head arthroplasty was done after dissecting the peroneal vessels proximal to bifurcation. The construct was placed in the joint and plated to the radial diaphysis. The capsule was closed after the arthroplasty. Fluoroscopy was used to verify the placement and range of motion (ROM).

The saphenous vein was harvested for the microvascular anastomosis secondary to lack of pedicle length to obtain anastomosis without undue stretch. The interposition graft was sewn end to side with the brachial artery above its bifurcation. The brachial artery was ischemic for a total of 30 minutes after which the flow was restored and confirmed by Doppler flow cytometry of the hand and through anastomosis. Couplers were used to anastomose the vein graft to the venae comitantes as well as the vein graft to the flap. The peroneal artery was sewn end to end with the saphenous graft. Both the skin and bone flaps showed appropriate blood flow. Two implantable Doppler probes were utilized distal to the arterial anastomosis beyond the bifurcation point to the skin paddle and bone. The skin flap was inserted to cover the bone and hardware. The remainder of the soft tissue defect was covered with a split thickness skin graft. The lower left leg was closed primarily, and the external fixator was reapplied.

**Figure 4:**
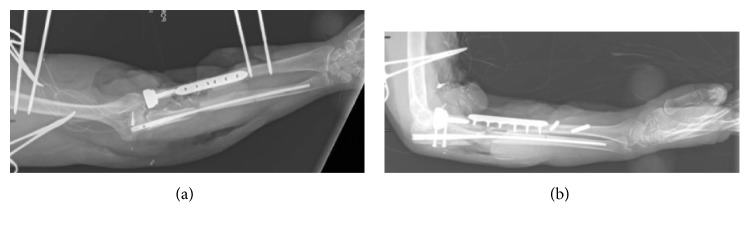
AP and lateral X-rays of the patient's forearm on POD 75. The ulnar has an IMN nail, and the radius has an open reduction internal fixation of the fibular free graft demonstrating good alignment.

The patient was transferred to the inpatient rehabilitation center within our institution on POD 7 from the osteocutaneous free flap transfer. On POD 30, the external fixator was removed. The patient had follow-up with both plastic surgery and orthopedic surgery. She had volar skin defect that closed secondarily of her right upper extremity. On POD 33, she was seen in clinic, observed passive ROM with limited restrictions on extension, flexion, pronation, and supination. With repeat X-ray of her extremity, she was shown to have appropriate interval consolidation with no loosening of hardware and good radial head placement. On POD75, the patient had difficulty extending her thumb and her fingers and the metacarpophalangeal joints secondary to the initial PIN injury. The range of motion around the elbow was 50 degrees for pronation/supination and 25–130 for flexion/extension. Her X-rays on POD 75 showed appropriate interval consolidation without loosening of hardware, good positioning of radial head, and appropriate interval healing of the ulnar fracture around the IMN nail, as seen in Figure 4. The Mayo Elbow Performance Index was 20 before the surgery, while it was 85 on POD 75. The patient had been stabilized to transfer back to her native country, so follow-up exams were limited. We had correspondence with her orthopedic surgeon abroad to convert the IMN of the ulna to a compression plate with bone grafting to encourage healing at the ulna nonunion site.

## 3. Discussion

Free vascularized flaps have revolutionized reconstructive surgery to restore cosmesis and functionality after debilitating injuries and large surgical defects. Fibular flaps are a common vascularized flap that includes both bone and subcutaneous tissue pedicles [7]. Many of the papers produced have demonstrated the graft being used to bridge the bone in the distal radial, proximal ulnar, and humeral reconstruction, but few have been used to restore joint function [5]. Scaglioni et al. demonstrated the use of vascularized bone grafts in 5 patients with restoration of elbow function: most of the injuries involved the distal humerus or proximal ulna [7]. While endoprosthetic reconstruction is the gold-standard for elbow injuries, the vascularized free flap has allowed for continued growth in pediatric populations, lower risk of infection and hardware malfunction, and lower risk of loosening of prosthesis, and in the setting of extensive bone loss, endoprosthetic repair is prohibitive [7, 8].

Functional range of motion around the elbow is necessary for daily living and hygiene and more intrinsic motions of contemporary society. The radiocapitellar joint carries most of the responsibility for forearm rotation (pronation/supination), and the ulnohumeral joint allows for flexion and extension [9]. Arcs of motion around the elbow joint require varying magnitudes, with previous studies demonstrating 130° of flexion and 100° of supination and pronation adequate for most activities of daily living [1].

ORIF has a limited role in radial head fractures where the fracture cannot be appropriately reduced, the articular congruity cannot be restored, or the elbow does not have adequate motion and stability [10]. One study demonstrated the fibular free flap and radial head arthroplasty in a patient with nonunion after pathologic fracture [5]. On one-year follow-up, the patient had active mobility of the elbow joint [5]. Allografts have also been used to bridge large bony defects in various resections requiring major debridement. One case report demonstrated successful allograft implantation after proximal radial head and neck fracture with satisfactory restoration of form and function [11]. Complications of allograft implantation consist of nonunion at the host-allograft junction, fractures, and infections [12, 13]. Meanwhile, fibular free transfer complications consist of longer operative periods of time, increased time to hypertrophy, donor site morbidity, and stress fractures [12]. Current studies have been conducted using allograft in combination with vascularized pedicle free flaps with comparable outcomes [13, 14]. More cases of radial head debridements and vascularized flaps have to be produced. This serves as an important modality for limb salvage and maintenance of elbow function.

The role of the fibular free graft has become an important treatment modality for upper extremity limb salvage for a variety of causes: cancers, osteomyelitis, intractable nonunion, and now even high-velocity traumas with extensive bony loss [2]. The patient in this case had been spared an amputation at initial presentation and, on follow-up, had a return of elbow function and healing of the joint. We propose that fibular osteocutaneous free flaps can be used as an appropriate operative conduit with the use of radial head arthroplasty to restore joint function after trauma.
